# Tuning Functionalized Ionic Liquids for CO_2_ Capture

**DOI:** 10.3390/ijms231911401

**Published:** 2022-09-27

**Authors:** Ruina Zhang, Quanli Ke, Zekai Zhang, Bing Zhou, Guokai Cui, Hanfeng Lu

**Affiliations:** College of Chemical Engineering, Zhejiang University of Technology, Huzhou 313299, China

**Keywords:** active site, functionalization, task-specific, separation, greenhouse gas control, CCUS, CO_2_-philic sorbent, carbon neutral, chemisorption, decarbonization

## Abstract

The increasing concentration of CO_2_ in the atmosphere is related to global climate change. Carbon capture, utilization, and storage (CCUS) is an important technology to reduce CO_2_ emissions and to deal with global climate change. The development of new materials and technologies for efficient CO_2_ capture has received increasing attention among global researchers. Ionic liquids (ILs), especially functionalized ILs, with such unique properties as almost no vapor pressure, thermal- and chemical-stability, non-flammability, and tunable properties, have been used in CCUS with great interest. This paper focuses on the development of functionalized ILs for CO_2_ capture in the past decade (2012~2022). Functionalized ILs, or task-specific ILs, are ILs with active sites on cations or/and anions. The main contents include three parts: cation-functionalized ILs, anion-functionalized ILs, and cation-anion dual-functionalized ILs for CO_2_ capture. In addition, classification, structures, and synthesis of functionalized ILs are also summarized. Finally, future directions, concerns, and prospects for functionalized ILs in CCUS are discussed. This review is beneficial for researchers to obtain an overall understanding of CO_2_-philic ILs. This work will open a door to develop novel IL-based solvents and materials for the capture and separation of other gases, such as SO_2_, H_2_S, NOx, NH_3_, and so on.

## 1. Introduction

According to the report “*State of the Global Climate 2021*” recently published by the World Meteorological Organization (WMO), CO_2_ mole fraction reached new high (413.2 ± 0.2 ppm in 2020, while pre-industrial mole fraction of 278 ppm [1]. The increasing concentration of CO_2_ in the atmosphere during these centuries, especially since the 20th century, leads to the greenhouse effect and global climate change. A large amount of all human-produced CO_2_ emissions come from the burning of fossil fuels, such as coal, natural gas, and oil, including gasoline.

In recent decades, carbon capture, utilization, and storage (CCUS) has become one of the important technologies to reduce CO_2_ emissions [2]. For carbon capture, the common CCUS technologies are based on chemical sorption, physical sorption, membrane separation, calcium looping, etc. For example, aqueous monoethanolamine (30 wt%) process is the current CO_2_ capture technology in industry via carbamate mechanism. Although the chemical reaction methods are more efficiency, the regeneration energy consumption of these methods is high [3]. For carbon utilization, the most effective strategy is CO_2_ convention, including thermocatalysis, photocatalysis, or electrocatalysis, of CO_2_ cycloaddition reaction, CO_2_ reduction reaction (CO_2_RR), etc. [4,5]. For example, the final products of the CO_2_RR are widely distributed from C_1_ (carbon monoxide, formic acid, methane) to C_2+_ (ethylene, ethanol, acetone, etc.) [6]. However, the CO_2_ conversion via CO_2_RR approach is still steps away from widespread commercialization. For carbon storage, the widely used way to store captured CO_2_ is in deep geological formations, such as oil fields, gas fields, coal seams, and saline aquifers [7]. However, the process increases the amount of energy required by power plants. Therefore, alternative CCUS technologies with high efficiency and low energy-consumption are highly desired.

Ionic liquids (ILs) are composed of organic cations and organic or inorganic anions with melting points below 100 °C [8,9,10,11]. Their excellent properties, including extremely low vapor pressure, high thermal and chemical stability, wide liquid temperature range, high electrical conductivity and wide electrochemical window, and good solubility for both polar and non-polar compounds, make it possible for ILs to be designed according to needs. Thus, ILs are widely used as green solvents and catalysts in such fields as energy and environment [12,13,14], chemistry and chemical synthesis [15,16], sorption and separation [17], and pharmaceutics and medicine [18,19]. Compared with conventional methods, ILs, especially functionalized ILs, have been used in CCUS with great interest all over the world due to the advantages of fast absorption, high capacity, low energy-consumption, good stability, and good recyclability.

Several interesting reviews for CO_2_ capture by ILs have been published during the last few years. For example, Zhang et al. [20] reviewed the IL-based CO_2_ capture systems from structure and interaction to process. Zhang and Ji et al. [21] reported the reviewing and evaluating of ionic liquids/deep eutectic solvents for CO_2_ capture. However, it is crucial to review this developing field from a viewpoint of functionalization of ILs with active sites, which is beneficial for researchers to obtain an overall understanding of CO_2_-philic ILs and grasp the development direction.

**Figure 1:**
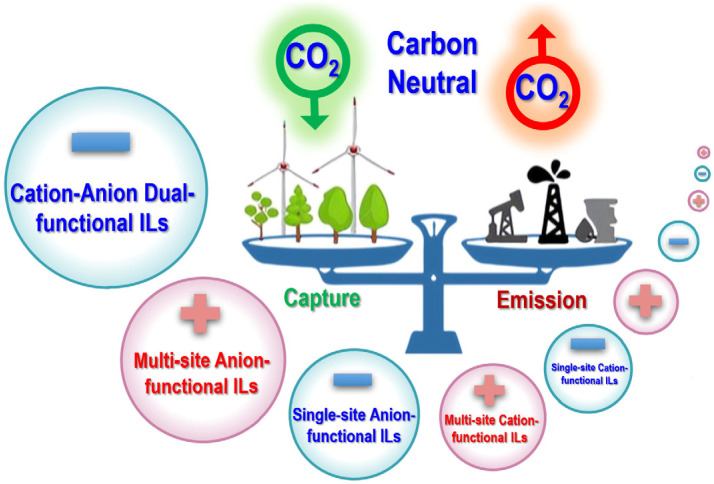
A summary of different kinds of functionalized ILs for CO_2_ capture.

In this critical review, we mainly focus on the development of functionalized ILs for CO_2_ capture in the past 10 years (2012~2022). The main contents include three parts, cation-functionalized ILs, anion-functionalized ILs, and cation-anion dual-functionalized ILs for CO_2_ capture (Figure 1). Besides, classification, structures, and synthesis of functionalized ILs are also summarized. Finally, future directions, concerns, and prospects for functionalized ILs in CCUS are discussed.

## 2. Classification, Structures, and Synthesis of Functionalized ILs

### 2.1. Classification and Structures of Functionalized ILs

**Figure 2:**
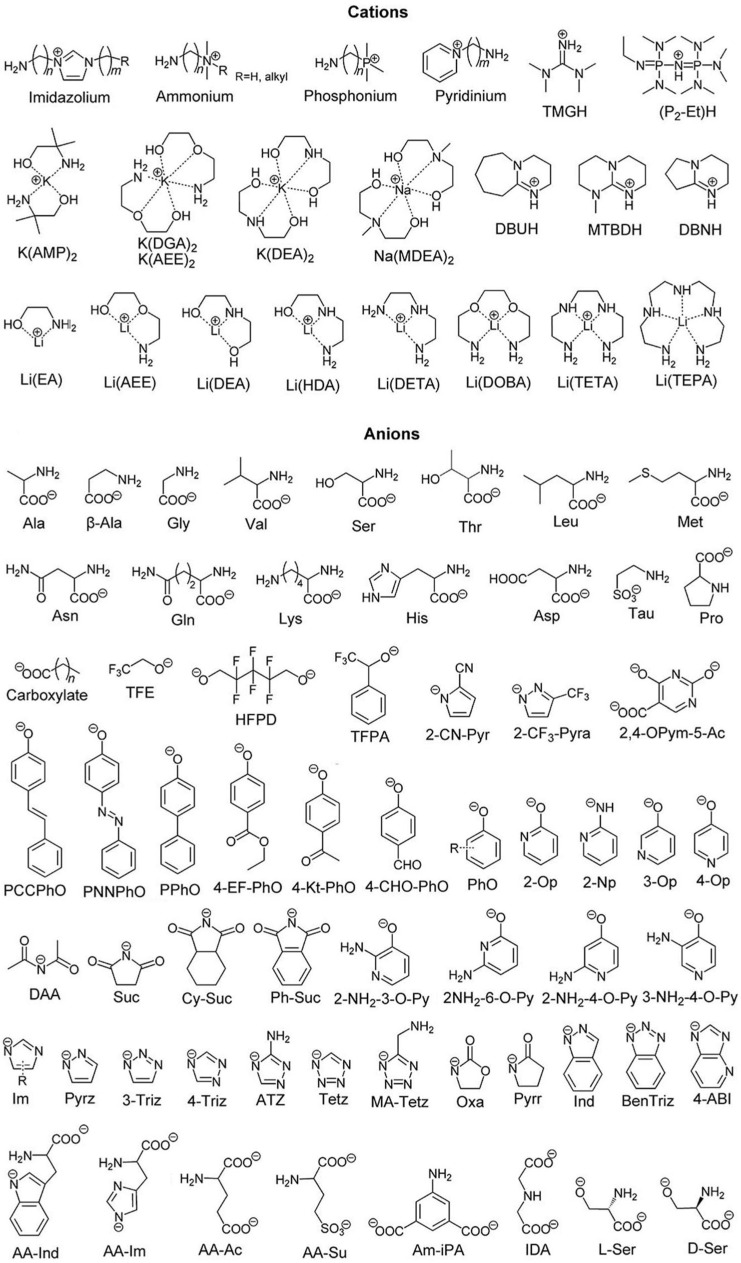
Structures of typical cations and anions used for designing functionalized ILs.

Functionalized ILs (task-specific ILs, or functional ILs) can be simply classified into three categories according to the locations of active sites, including cation-functionalized ILs, anion-functionalized ILs, and cation-anion dual-functionalized ILs. The cation-functionalized ILs and anion-functionalized ILs can be divided into two categories according to the number of functional groups and the mechanism of CO_2_ capture, including single-site functionalized ILs and multiple-site functionalized ILs. It is clear that cation-anion dual-functionalized ILs are multiple-site functionalized ILs. The main reaction groups with active sites are listed in each category, such as amino, carboxylate, alkoxide, phenolate, and azolate. The structures of cations and anions for synthesis of functionalized ILs are collected in Figure 2.

### 2.2. Synthesis of Functionalized ILs

**Figure 3:**
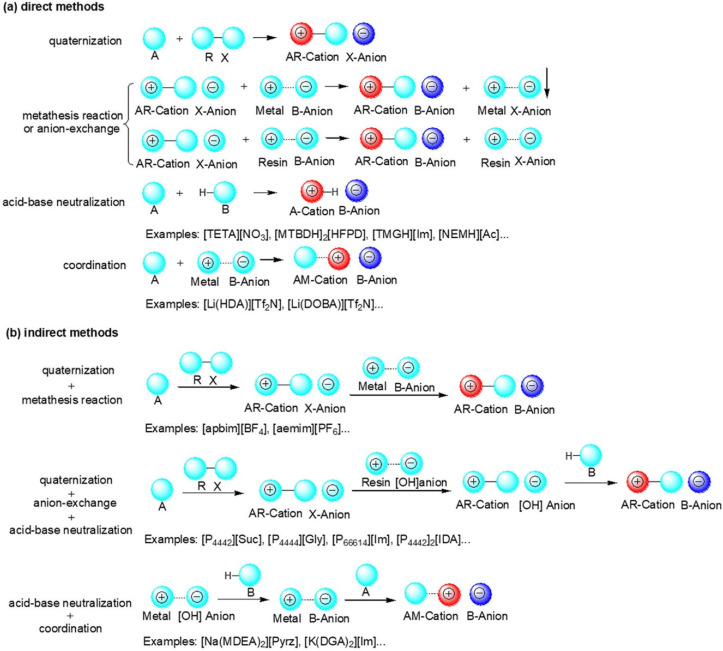
Typical (**a**) direct methods and (**b**) indirect methods for synthesis of functionalized ILs for CO_2_ capture.

Generally, the synthesis of functionalized ILs includes several separated unit operations, such as quaternization, anion-exchange, acid-base neutralization, coordination, etc. According to the structure of functionalized ILs, the methods or pathway for the synthesis can be typically classified into two categories: direct methods and indirect methods. Direct methods include one of above-mentioned operations, while indirect methods include two or more of above-mentioned operations. The typical strategies for synthesis of functionalized ILs for CO_2_ capture can be found in Figure 3.

## 3. Functionalized ILs for CO_2_ Capture

### 3.1. Cation-Functionalized ILs for CO_2_ Capture

#### 3.1.1. Single-Site Mechanisms

**Figure 4:**
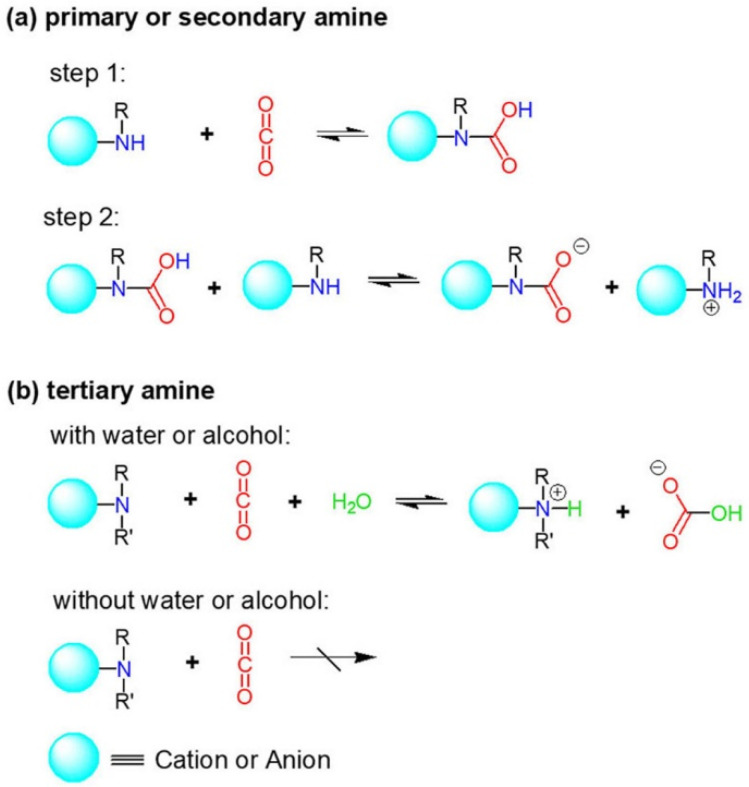
General mechanisms of amino-CO_2_ reactions for (**a**) primary or secondary amine, and (**b**) tertiary amine.

It is known that the most studied cation-functionalized ILs for CO_2_ capture should be amino-functionalized ILs, which were first reported by Davis et al. [22] in 2002, two decades ago. They showed that 0.5 mole of CO_2_ per mole of IL was captured by 1-propylamide-3-butyl imidazolium tetrafluoroborate ([apbim][BF_4_]) via a carbamate mechanism (2 amino: 1 CO_2_). Compared with conventional alkanolamine aqueous solution (30 wt% monoethanolamine) for CO_2_ capture, amino grafted on cations of ILs showed high thermostability [23,24], while amino grafted on ILs showed high capture capacity compared with conventional ILs [25]. Subsequently, a number of amino-grafted cation-functionalized ILs were reported for efficient CO_2_ capture [26,27,28,29,30]. The mechanisms of amino–CO_2_ reaction in ILs are similar to those in aqueous alkanolamine solutions. Compared with primary and secondary amines, tertiary amine is considered unreactive with CO_2_ under anhydrous conditions (Figure 4). However, He et al. [31] reported that tertiary amino-containing Li-chelated cation-functionalized ILs, [PEG_150_MeBu_2_NLi][Tf_2_N] and [PEG_150_MeTMGLi][Tf_2_N], could achieve high CO_2_ capacities, 0.66 and 0.89 mol CO_2_ per mol IL, respectively, via coordination with lithium ion.

#### 3.1.2. Multiple-Site Mechanisms

Multiple functional sites on the cations are multiple amino groups. For example, Zhang et al. [32] reported CO_2_ capture by a dual amino-containing cation-functionalized IL, 1, 3-di (2′-aminoethyl)-2-methylimidazolium bromide (DAIL), via a 2:1 carbamate mechanism (amino: CO_2_). However, the synthesis of DAIL was not easy. Therefore, other kinds of polyamine-based ILs were developed through acid-base neutralization or metal coordination. Clyburne et al. [33] and Meng et al. [34] studied CO_2_ capture by [DETA][NO_3_] and [TETA][NO_3_] ammonium ILs, which were prepared through acid-base neutralization of diethylenetriamine (DETA) or triethylenetetramine (TETA) with nitric acid. On the other hand, Wang and Dai et al. [35] investigated the CO_2_ capture by a series of chelate ILs with multiple Li-coordinated amino groups on the cations, and up to 0.88 and 0.90 mol CO_2_ per mol IL could be captured by [Li(HDA)][Tf_2_N] and [Li(DOBA)][Tf_2_N] at 40 °C and 1 bar, respectively, via a 2:1 mechanism. Subsequently, Wang et al. [36] reported several polyamine-based ILs ([Li(TETA)][Tf_2_N], [Li(DETA)][Tf_2_N] and [Li(TEPA)][Tf_2_N]) and polyalcohol-based ILs ([Li(TEG)][Tf_2_N] and [Li(TTEG)][Tf_2_N]). The former could chemically absorb CO_2_, while the later could only physically absorb CO_2_. Their results showed that CO_2_ capacity of polyamine-based ILs increased when [Li(TTEG)][Tf_2_N] or [Li(TEG)][Tf_2_N] was added, and CO_2_ capacity of [Li(TEPA)][Tf_2_N]/[Li(TEG)][Tf_2_N] (weight ratio is 1:2) decreased from 2.05 to 0.83 mol per CO_2_ mol IL at 80 °C when CO_2_ concentration was reduced from 100 vol.% to 380 ppm. Recently, Yang, Xing, and Dai et al. [37] reported the tuning of stability constants of metal-amine complexes for efficient CO_2_ desorption.

The comparison of the absorption capacities, including molar capacities and corresponding mass capacities, of typical cation-functionalized ILs for CO_2_ capture are listed in Table 1.

### 3.2. Anion-Functionalized ILs for CO_2_ Capture

Compared with only amino-grafted cations for efficient CO_2_ capture by ILs, there are numerous kinds of functional groups grafted on anions for efficient CO_2_ capture. According to their reaction mechanism with CO_2_, the anion-functionalized ILs can be classified into two categories, including single-site mechanisms and multiple-site mechanisms.

#### 3.2.1. Single-Site Mechanisms

**Figure 5:**
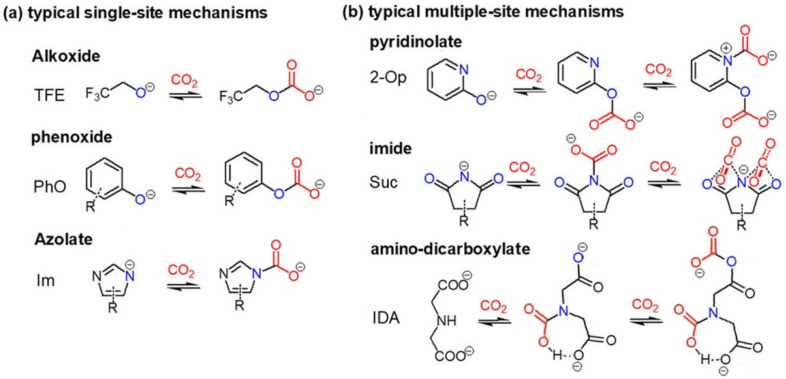
Typical (**a**) single-site and (**b**) multiple-site mechanisms of non-amino anion-CO_2_ reactions.

Functionalized ILs with single-site on anions include amino anions, carboxylate anions, alkoxide anion, phenolate anions, and azolate anions. The typical mechanisms for the reaction of non-amino anion-CO_2_ can be found in Figure 5a.

(1) Amino anion functionalized ILs

Typical amino anions are amino acid anions that are prepared via dehydrogenation (or acid-base neutralization). For example, several amino acid ILs (AAILs) ([P_4444_][Gly], [P_4444_][Ala], [P_4444_][β-Ala], [P_4444_][Ser], and [P_4444_][Lys]) with high viscosity were first reported by Zhang et al. [38] for CO_2_ capture through supporting on SiO_2_, and the absorption following a 2:1 carbamate pathway. However, [P_66614_][Met] and [P_66614_][Pro] with large phosphonium cations were reported by Brennecke et al. [39,40] for equimolar absorption of CO_2_ via a 1:1 mechanism. In order to understand the CO_2_ absorption mechanisms with AAILs, Xing et al. [41] showed that the actual mechanism went beyond the apparent stoichiometry. Take [Gly] and [Met] anions as the examples, although the apparent chemical stoichiometry approached 1:1 and the absorption was previously considered to simply follow the 1:1 mechanism, their results indicated that more than 20 % of the CO_2_ still was absorbed in the 1:2 reaction mechanism. Recently, Mehrdad et al. [42] investigated three AAILs ([BMIm][Gly], [BMIm][Ala], and [BMIm][Val]) for CO_2_ capture via physical and chemical sorption mechanism. However, the hydrogen bond in these AAILs resulted in high viscosity, and the viscosity increased dramatically after the absorption of CO_2_. Therefore, other AAILs supported on porous materials [43,44,45,46] or mixed with liquids [33,34] were reported;

(2) Carboxylate anion-functionalized ILs (O-site)

From the 1:1 mechanism of AA ILs with CO_2_, the carboxylate in the AA anions provides a negative charge but seemed to not interact with CO_2_. Through tuning the structure of carboxylate ILs, Ils can also chemically react with CO_2_. 1-Butyl-3-methylimidazolium acetate ([Bmim][Ac]), reported by Maginn et al. [47], was the first carboxylate IL example for efficient CO2 capture. The reported mechanism of N-heterocyclic carbene−CO2 was verified by NMR. However, Steckel et al. [48], Shi et al. [49], and Ruiz-López et al. [50] studied the mechanism via ab initio calculations. The results indicated that for glycinate anion, interactions with the amino and carboxylic moieties involved comparable energetics. For example, Tao et al. [51] studied a series of phosphonium carboxylate ILs for CO2 capture, and butyrate IL could absorb 0.4 mol CO_2_ per mol IL. Yunus et al. [52] investigated ammonium carboxylate ILs with different organic acid anions for CO_2_ capture at high pressures and obtained the high capacities of ILs with heptanoate anions. Cheng et al. [53] correlated the data of CO_2_ solubility in carboxylate-based N-ethylmorpholinium ILs ([NEMH][Ac], [NEMH][Propionate], and [TEAH][Propionate]) with Pitzer’s model and the Soave–Redlich–Kwong cubic equation of state. Similarly, Umecky et al. [54] showed that acetylacetonate ILs could also chemically absorb CO_2_;

(3) Alkoxide anion-functionalized ILs (O-site)

The alkoxide is an anion that forms when we remove the hydrogen atom from the –OH group of an alcohol. It was known that switchable solvents, a liquid mixture of an alcohol (e.g., pKa of ethanol in DMSO is 29.8) and a strong organic base (e.g., 1,8-diazabicyclo-[5.4.0]undec-7-ene, DBU), could chemically bind CO_2_ to form an alkylcarbonate salt through proton transfer from alcohol to superbase [55,56]. Thus, alcohols with the appropriate acidity can be used to synthesize alkoxide ILs through dehydrogenation. Dai et al. [57] reported a series of superbase-derived protic ILs with trifluoroethanolate (TFE, pKa = 23.5), 1-phenyl-2,2,2-trifluoroethanolate (TFPA, pKa = 23), and 2,2,3,3,4,4-hexafluoro-1,5-pentanediolate (HFPD, pKa = 23.2) anions for equimolar CO_2_ capture. Subsequently, Liu et al. [58] used [DBUH][TFE] (1.01 mol CO_2_ per mol IL) to catalyze CO_2_ conversion into quinazoline-2,4(1H,3H)-diones;

(4) Phenolate anion-functionalized ILs (O-site)

With appropriate acidity (pKa = 10 in water), the phenol could be used to form the functional anion, phenolate (or phenoxide), via removal of the hydrogen atom from the –OH group of a phenol to prepare ILs for efficient CO_2_ capture. For example, Wang et al. [59] studied a series of phenolate anion-functionalized ILs for CO_2_ chemisorption. Through tuning the structure of phenolate anion with different substituents, CO_2_ absorption performance could be further regulated. For example, the absorption capacities of [P_66614_][4-Me-PhO], [P_66614_][4-H-PhO], [P_66614_][4-Cl-PhO], [P_66614_][4-CF_3_-PhO], [P_66614_][4-NO_2_-PhO], and [P_66614_][2,4,6-Cl-PhO] were found to be 0.91, 0.85, 0.82, 0.61, 0.30, and 0.07 mol CO_2_ per mol IL, respectively. The same authors also found carbonyl-substituted phenolate ILs, [P_66614_][4-Kt-PhO], [P_66614_][4-EF-PhO], and [P_66614_][4-CHO-PhO] could achieve 1.04, 1.03, and 1.01 mol CO_2_ per mol IL at 20 °C and 1 bar, respectively [60]. Additionally, they also synthesized a series of conjugated phenolate ILs and investigated their CO_2_ absorption performance [61]. The results showed that the molar ratios of CO_2_ to [P_66614_][PPhO] and [P_66614_][PCCPhO] were 0.93 and 0.96, respectively. Wu and Hu et al. [62,63] investigated that fluorinated phenolate ILs with different positions resulted in low viscosity and tunable capacity ([4-F-PhO] > [3-F-PhO] > [2-F-PhO]). Recently, Yang, Xing, and Dai et al. [64] constructed several phenolate chelate ILs for CO_2_ chemisorption via coordination of phenolate alkali metal salts with crown ethers. With 15-crown-5-coordinated Na^+^ as the cation, the CO_2_ uptake capacity of the phenolate anion decreased in the following order: [PhO]^−^ (0.75 mol mol^−1^) > [*n*-C_3_H_7_PhO]^−^ (0.66 mol mol^−1^) > [*n*-C_8_H_17_PhO]^−^ (0.50 mol mol^−1^);

(5) Azolate anion-functionalized ILs (N-site)

Azoles, such as imidazole (Im), pyrazole (Pyrz), 1,3,4-trizole (Triz), tetrazole (Tetz), etc., are a kind of five-membered heterocycles. These azoles with high basicity were used by Dai and Wang et al. [57,65] for preparing anion-functionalized ILs via dehydrogenation for CO_2_ capture. There have been numerous investigations on CO_2_ capture by azolate ILs, including protic ILs and aprotic ILs, during this decade. Take imidazolate ILs as an example, the reported CO_2_ absorption capacities of [MTBDH][Im] [57], [(P_2_-Et)H][Im] [57], [P_66614_][Im] [65,66], [TMGH][Im] [67,68], [DBUH][Im] [69,70,71], and [DBNH][Im] [70] were in the rage of 0.8~1.0 at room temperature and atmospheric pressure. Different from aprotic cations or protic cations with a strong base, Oncsik and MacFarlane et al. [72] and Yang et al. [73] reported N,N-dimethylethylenediaminium azolate ILs for CO_2_ capture via forming carbamate species. The former authors believed that CO_2_ reacted with azolate anions, while the latter authors found that CO_2_ reacted with the cations. Thus, it is well understood that (1) the anion but not the cation has the key function in CO_2_ capture and (2) the mechanism follows a 1:1 stoichiometry. On the other hand, the absorption molar ratios of CO_2_ to IL were affected by the basicity of ILs. Wang et al. [65] showed that the absorption capacity decreased from 1.02 for [P_66614_][Pyrz] to 0.08 for [P_66614_][Tetz], when the pKa value of the azoles decreased from 19.8 for Pyrz to 8.2 for Tetz, and [P_66614_][Triz] was an ideal IL with desirable absorption enthalpy (−56 kJ mol^−1^) and high absorption capacity (0.95 mol CO_2_ per mol IL). Subsequently, kinds of [Triz]-based ILs were reported for CO_2_ capture [74,75].

The substituents on the heterocycles will affect the performance of CO_2_ capture by azolate ILs. Wu et al. [76] studied the reactivity of azolate anions with CO_2_ from the density functional theory (DFT) perspective. It was studied that the absorption capacity by imidazolate [66,69], pyrazolate ILs [77,78], and indazolate ILs [79] was affected by the substituents on the heterocycles. For example, Wang et al. [60] showed that the molar ratio of CO_2_ to [P_66614_][4-CHO-Im] could reach 1.24 at 20 °C and 1 bar via the interactions of imidazolate-CO_2_ and hydrogen bonding. Later, they also concluded that [P_66614_][4-Br-Im] was an ideal substituent imidazolate IL with desirable absorption enthalpy (−61.4 kJ mol^−1^), basicity (pKa is 12.2 in H_2_O), and CO_2_ capacity (0.87 mol CO_2_ per mol IL) [66]. It is also clearly that the substituents resulted in different basicity of azolate ILs. Recently, a light-responsive 1,3,4-trizolate IL was reported by Wang et al. [80], and the decreased capacity of CO_2_ was found when the IL converted from the *trans* to *cis* state. They confirmed that the entropy change was the key influencing factor. Additionally, different from the amino-functionalized ILs with the viscosity increasing during the CO_2_ absorption, the viscosity of the viscosity of [P_66614_][Im] was found to decrease from 810.4 cP to 648.7 cP after absorption of CO_2_. Jiang et al. [81] revealed the microscopic origin for the decrease in viscosity after CO_2_ absorption by [P_66614_][Im] via molecular dynamics (MD) simulation. Rogers et al. [76] reviewed the ILs with azolate anions due to their desired properties, including a diffuse ionic charge, tailorable asymmetry, and synthetic flexibility. Azolate anion-functionalized ILs are also reported with the name “aprotic heterocyclic anion” ([AHA]) based ILs. Maginn et al. [82] reported [P_66614_][2-CN-Pyr]) and [P_66614_][2-CF_3_-Pyra] could obtain ~0.9 mol CO_2_ per mol IL via a 1:1 mechanism. Brennecke et al. [83,84] investigated the influence of substituent groups on the reaction enthalpy of CO_2_–[AHA], and the estimated values range between −37 and −54 kJ mol^–1^, lower than that of CO_2_–MEA (−85 kJ mol^–1^). The structure and mechanism of azolate–CO_2_ was systematically studied via DFT calculations, [82] ab initio MD simulation [85,86,87], Monte Carlo simulation [88], first principles simulations [89,90], and other computer calculations [91,92].

Another reaction pathway was reported. It should be noted that when the anion has a certain basicity for CO_2_ capture, the basicity of the anion can cause it to pull out an active hydrogen atom on the imidazolium or phosphonium cation to form a carbene or zwitterionic compound (ylide), which could subsequently interact with CO_2_ to form a carbene–CO_2_ or ylide–CO_2_ complex, respectively. Brennecke et al. [93] selected four azolate ILs with different basicity and low basicity for [Tetz] and high basicity for [3-Triz], [4-Triz], and [2-CN-Pyr]. They quantified the amounts of cation–CO_2_ and anion–CO_2_ complexes. For [Emim][2-CN-Pyr], 59% of the C2 acidic protons are removed, leaving the carbene to react with CO_2_ and form the cation–CO_2_ complex. Chen et al. [94] showed that the more basic [AHA] anion would form carbene–CO_2_ via DFT. As the formed carbene–CO_2_ resulted in the reduced efficiency of anions, Wang et al. [95] investigated that substituted imidazolium reduced the amount of carbene–CO_2_ and increased the amount of azolate–CO_2_. For the ylide–CO_2_ pathway, Brennecke et al. [78,96] investigated the cation–anion and [AHA] –CO_2_ interactions and the quantification of ylide–CO_2_ in phosphonium ILs. In addition to the azolate anions, the phenolate anions and carboxylate anions can also result in carbene–CO_2_ in imidazolium ILs [97,98,99] or ylide–CO_2_ in phosphonium ILs [100], respectively.

The comparison of the absorption capacities, including molar capacities and corresponding mass capacities, of typical anion-functionalized ILs for CO_2_ capture via single-site mechanisms are listed in Table 2.

#### 3.2.2. Multiple-Site Mechanism

It is known that single-site in ILs result in up to a 1:1 stoichiometry absorption capacity. However, multiple-site in ILs may not result in doubled capacity. For multiple sites sharing one negative charge, the efficiency of a site may be decreased. Besides, even if the two sites are independent or each has a negative charge, the absorption capacity may not double, due to the complex interactions in ILs. The typical multiple-site mechanisms can be found in Figure 5b.

(1) Multiple same groups in anion-functionalized ILs

As amino group is a functional group for CO_2_ capture, AAILs based on amino acid anions with multiple amino were developed, including [Lys], [His], [Asn], and [Gln]. For [Lys], the molar ratios of CO_2_ to [P_66614_][Lys] [101], [N_66614_][Lys] [102], [C_2_OHmim][Lys] [103], and [N_1,1,6,2O4_][Lys] [104] were 1.37, 2.1, 1.68, and 1.62, respectively, via the reaction mechanism of 1:1. Different from two amino groups in one anion, CO_2_ capacities of several dicationic ILs with two amino acid anions [105] or azolate anions [106,107] were reported nearly twice that of the monocationic analogues. Additionally, CO_2_ absorption capacity of a superbase-derived diolate IL [MTBDH]^+^_2_[HFPD]^2−^ reported by Dai et al. [57] was more than 2.04 mol CO_2_ per mol IL because of two alkoxide groups. Wang et al. [108] investigated the CO_2_ capture by a pillar[5]arene-based −10 valent carboxylate anion-functionalized phosphonium IL, [P_66614_]_10_[DCP5]. Their results showed that capacities of 5.52 mol CO_2_ per mol IL and 0.55 mol CO_2_ per mol carboxylate could be obtained through multiple-site interactions;

(2) Pyridinolate anion-functionalized ILs

Although the N atom in neutral pyridine has poor ability for CO_2_ capture [109], Wang et al. [110] reported CO_2_ capture by a series of hydroxypyridine-based anion-functionalized ILs, including [P_66614_][2-Op], [P_66614_][4-Op], [P_66614_][3-Op], etc. The CO_2_ capacities of these ILs were more than 1 (up to 1.65) mol CO_2_ mol^−1^ IL due to the cooperative N–CO_2_ and O–CO_2_ interactions. Hao and Guan et al. [111] investigated the anion–CO_2_ interaction in hydroxypyridinate ILs with [P_4444_] or [N_4444_] cations via quantum chemistry calculations. A viscosity as low as 193 cP with an absorption capacity as high as 1.20 mol CO_2_ per mol IL were obtained by [P_4444_][2-Op]. Lin and Luo et al. [112] investigated a series of hydroxypyridine ILs with different kinds of cations, and the enhanced CO_2_ capacity up to 1.83 mol CO_2_ mol^−^^1^ IL could be obtained at 20 °C and 1 bar via reducing cation-anion interactions. Xu et al. [113] reported that the CO_2_ capture capacity of ILs with [DBUH] and [TMGH] cations and hydroxypyridine anions followed the order of [2-Op]^−^ > [4-Op]^−^ > [3-Op]^−^. The molar ratio of CO_2_ to [DBUH][2-Op] at 40 °C was up to ~0.90 mol CO_2_ per mol IL, similar to azolate ILs. In order to enhance the adsorption kinetics, CO_2_ capture by [2-Op]-based ILs were performed on porous supports with high capacities [114,115];

(3) Imide anion-functionalized ILs

Imide and amide anions reported to have nucleophilic reactivities [116]. In order to improve CO_2_ capacity under low concentration CO_2_ (10 vol%), Cui and Wang et al. [117] synthesized a series of imide anion-functionalized ILs, [P_4442_][Suc] and [P_4442_][DAA]. Through pre-organization strategy, the prepared [P_4442_][Suc] showed a high efficiency on CO_2_ capture (1.65 mol CO_2_ mol^−1^ IL for 10 vol% and 1.87 mol CO_2_ mol^−1^ IL for 100 vol%) via cooperative 3 site (O-N-O)-2–CO_2_ interaction. Further studies showed that the electro-withdrawing phenyl group on the anion, [Ph-Suc], reduced the CO_2_ absorption capacity, while the electro-donating cyclohexyl group on the anion, [Cy-Suc], increased the CO_2_ absorption capacity (1.76 mol CO_2_ mol^−1^ IL for 10 vol% and 2.21 mol CO_2_ mol^−1^ IL for 100 vol%) via enhanced cooperation and physical interaction [118]. Additionally, the obtained imide-based ILs are stable in water, and the CO_2_ absorption could be improved under low water content [119]. The results of thermodynamic studies showed that the absorption was an enthalpy-driven process[120]. Wang et al. [121] reported an aminomethyl-functionalized tetrazolate IL, [P_66614_][MA-Tetz], with a CO_2_ capacity of 1.13 mol CO_2_ per mol IL, due to the interaction of one amino group (H-N-H) with two molecules of CO_2_;

(4) Other multiple-site anion-functionalized ILs

When multiple sites are independent in an anion, they give an opportunity for improving CO_2_ capture. As amino and carboxylate could both interact with CO_2_ efficiently, Tao et al. [122] synthesized the [P_4442_]_2_[IDA] with a −2 valent amino acid anion, and the improved absorption capacity was 1.69 mol CO_2_ per mol IL through amino-CO_2_ and carboxylate-CO_2_ interactions. Subsequently, Pan and Zou et al. [123] reported a series of AA ILs based on −2 valent amino acid anions. Compared with hydroxyl-containing −1 valent counterparts, alkoxide anion-containing −2 valent AA ILs, [P_4442_]_2_[D-Ser] and [P_4442_]_2_[L-Ser], showed high CO_2_ capacity due to the interactions of amino-CO_2_ and alkoxide-CO_2_. Luo and Lin et al. [124] reported kinds of −2 valent AA ILs, [P_66614_]_2_[AA-R], where R was sulfonate (Su), carboxylate (Ac), imidazolate (Im), or indolate (Ind). The CO_2_ capacities of [P_66614_]_2_[AA-Su], [P_66614_]_2_[AA-Ac], [P_66614_]_2_[AA-Im], and [P_66614_]_2_[AA-Ind] were 0.49, 1.97, 1.55, and 1.45 mol CO_2_ per mol IL, respectively. The results presented that CO_2_ capacity increased first and then decreased later with the continuous increase in the activity of the anion site.

On the other hand, when multiple sites are dependent in an anion, their interactions may lead to mutual restraint for CO_2_ capture. For example, Liu et al. [125] synthesized a -3 valent carboxylate- hydroxypyridinate- containing anion IL, [P_4444_]_3_[2,4-OPym-5-Ac] and found that a CO_2_ capacity of 1.46 mol CO_2_ per mol IL could be obtained, lower than the theoretical value. However, Luo and Lin et al. [124] reported a −2 valent IL, [P_66614_]_2_[Am-iPA], with amino functionalized dicarboxylate anion. Their results showed that CO_2_ capture capacity of this IL was 2.38 mol CO_2_ per mol IL at 30 °C. Besides, multiple sites dependently shared one negative charge, resulting in decreased efficiency of CO_2_ capture. Wang and MacFarlane et al. [126] studied CO_2_ capture by an amino-containing hydroxypyridinate anion-functionalized ILs with the capacity of 0.87~0.99 mol CO_2_ per mol IL. The NMR results indicated the primary reaction of amino-CO_2_ and the lesser reaction of phenolate-CO_2_. Tao et al. [127] reported CO_2_ capture by amino-functionalized triazolate anion ILs, [Bmim][ATZ] and [Emim][ATZ], with a capacity as low as 0.14 and 0.13 mol CO_2_ per mol IL, respectively, via physical interaction.

The comparison of the absorption capacities, including molar capacities and corresponding mass capacities, of typical anion-functionalized ILs for CO_2_ capture via multiple-site mechanisms are listed in Table 3.

### 3.3. Cation-Anion Dual-Functionalized ILs for CO_2_ Capture

It is clear that functional groups on cations are mainly amino groups. Thus, with the combination of functional cations and functional anions, kinds of dual-functionalized ILs with multiple sites were developed for CO_2_ capture.

#### 3.3.1. Amino-Based Cation and Amino-Based Anion

The early dual-functionalized ILs were ILs with amino-functionalized cations with amino acid anions. For example, [aP_4443_][AA] (Gly, and [Ala]) [128], [aemmim][Tau] [129], [apaeP_444_][AA] ([Lys], [Gly], [Ser], [Ala], [Asp], and [His]) [130], and [AEMP][AA] ([Gly], [Ala], [Pro], and [Leu]) [131], and [APmim][AA] ([Gly] and [Lys]) [132,133] were found to capture CO_2_ via 2:1 mechanism (amino:CO_2_). Jing et al. [134] used quantum chemical simulation for screening of multi-amino-functionalized ILs for CO_2_ capture. Their experimental results confirmed the predictions and the absorption capacities of [TETAH][Lys] (5 amino groups) and [DETAH][Lys] (4 amino groups) were 2.59 and 2.13 mol CO_2_ per mol IL, respectively, via 2:1 zwitterionic mechanism.

#### 3.3.2. Amino-Based Cation and Phenolate Anion

Based on the high reactivity of phenolate anions for CO_2_ capture, Ye and Li et al. [135] reported that the CO_2_ absorption capacities of supported dual functionalized phosphonium ILs [aP_4443_][2-Op] and [aP_4443_][2-Np] were 1.57 and 1.88 mol CO_2_ per mol IL, respectively, via 2:1 mechanism of amino-CO_2_ and 1:1 mechanism of phenolate-CO_2_ mechanism. Recently, Wang et al. [136] reported two dual-functionalized protic ILs, dimethylethylenediamine 4-fluorophenolate ([DMEDAH][4-F-PhO]) and dimethylethylenediamine acetate ([DMEDAH][OAc]) to investigate the different chemisorption mechanisms via DFT study. Their results showed that, for [DMEDAH][4-F-PhO], phenolate-CO_2_ was favorable in kinetics and amino-CO_2_ was thermodynamically beneficial; for [DMEDAH][OAc], amino-CO_2_ was favorable with proton-transfer to weak acid anion.

#### 3.3.3. Amino-Based Cation and Azolate Anion

Based on the high reactivity of azolate anions for CO_2_ capture, Ye and Li et al. [135] reported that the CO_2_ absorption capacities of supported dual functionalized phosphonium IL [aP_4443_][Triz] was 1.32 mol CO_2_ per mol IL via 1:1 mechanism of azolate-CO_2_ mechanism. Considering the metal coordination of amino groups as well as the CO_2_-philic azolate anions, Xu et al. reported a series of polyamine-based dual-functionalized azolate ILs with different structures of polyamines, metal ions, and azolate anions. For example, CO_2_ capacities of [Na(MDEA)_2_][Pyrz] [137], [K(DGA)_2_][Im] [138], and [K(AMP)_2_][Im] [139], were 0.75 (80 °C), 1.37 (60 °C), and 1.19 (60 °C) mol CO_2_ per mol IL, respectively, via reactions of amino-CO_2_ and azolate-CO_2_.

The comparison of the absorption capacities, including molar capacities and corresponding mass capacities, of typical cation-anion dual-functionalized ILs for CO_2_ capture are listed in Table 4.

## 4. Conclusions and Outlook

It is known that functionalized ILs started in 2002, and it has been just two decades. Due to the designable and tunable structures of ILs, functionalized ILs have developed rapidly in the past ten years (2012–2022). CO_2_-philic active sites can be tethered to the cations and anions, forming cation-functionalized ILs, anion-functionalized ILs, and cation-anion dual-functionalized ILs. Compared with conventional ILs for physisorption of CO_2_, functionalized ILs or task-specific ILs could chemically absorb CO_2_ through single-site mechanisms or multiple-site mechanisms. Based on the research results, we can safely conclude that efficient absorption of CO_2_ with a high capacity, low energy consumption, and high reversibility could be reached through tuning the structures of functionalized ILs and regulating the interactions between active sites and CO_2_. Nonetheless, for large-scale industrial application of IL-based CCUS technology, we also need to consider the following issues:

(1) Reaction mechanism of functionalized IL-CO_2_ needs to be investigated further;
(2) A large amount of CO_2_ absorption experiments was tested at room temperature and atmospheric pressure, but the temperature of flue gas is high (50~80 °C) and the concentration of CO_2_ is low (10~15 vol%), there is still a big gap between laboratory research and industrial application;
(3) The selective capture of CO_2_ and the deactivation of functionalized ILs under other gases conditions (H_2_O, SO_2_, NOx, etc.) should be studied;
(4) Compared with conventional absorbents such as alkanolamine aqueous solutions, pure functionalized ILs have higher viscosity and cost;
(5) It is important to investigate capture efficiency in mass absorption capacity or gravimetric capacity in order to better comparison and realize the competitive ILs. Thus, functionalized ILs with high mass absorption capacity should be developed.
(6) The regeneration of the ILs is also important and related to energetic consume and the absorption cost. Thus, the absorption enthalpies should be investigated.

Here are some suggestions or strategies to address the aforementioned issues:

(1) A combination of NMR and IR analysis and chemical calculations can be used to investigate the absorption mechanisms of active sites on the ILs with CO_2_;
(2) The performance of CO_2_ capture is affected by absorption temperature and CO_2_ partial pressure. Due to the tunable structure and property of ILs, design functionalized ILs with high active sites is an efficient way to help ILs applicate in industry;
(3) H_2_O, SO_2_, NOx, etc. will lead to a decrease in the activity of ILs, especially ILs with strong basicity. Thus, these impurities should first be removed. For example, ILs with weak basicity for SO_2_ or NOx removal and ILs with strong basicity for CO_2_ removal;
(4) Functionalized ILs with a low viscosity could be synthesized through tuning the structures of cation and anion. Besides, the viscosity of amine-containing functionalized ILs or protic ILs were reported to be increased during the absorption of CO_2_, while for amine-free functionalized ILs and aprotic ILs no obvious change during CO_2_ capture was reported due to the absence of strong hydrogen bonded networks in these ILs (Table 5);
(5) Aqueous monoethanolamine (30 wt%) process is the current CO_2_ capture technology in industry with a mass capacity of ~7 wt%. It can be found in Table 1, Table 2, Table 3 and Table 4 that functionalized ILs with a high molecular weight resulted in a high molar capacity but a low mass capacity. Functionalized ILs with a high molar capacity open the door to developing functionalized ILs with a high mass capacity via combining functional sites and a small molecular weight;
(6) High regeneration or reversibility of the ILs for CO_2_ capture needs weak interactions or low absorption enthalpies, which always results in low efficiency. Thus, functionalized ILs is always accompanied by high energy consumption. However, the results from CO_2_ capture by preorganized imide-based ILs indicate that multiple weak interactions also lead to strong adsorption and high capacity, even under low concentrations of CO_2_.

Therefore, continuously developing novel functional IL-based CO_2_-philic solvents or sorbents and systematically studying the reaction mechanism of CO_2_ with active sites under different conditions are the main concern of IL-based CCUS technologies in order to realize large-scale, rapid, economical, efficient, and reversible absorption of CO_2_ in the flue gas.

## Figures and Tables

**Table 1:** Typical cation-functionalized ILs for CO_2_ capture.

IL	*T* (°C)	*P* (bar)	*M*_w_ (g mol^–1^) *^a^*	*n* _CO_2__/*n* _IL_	*n* _CO_2__/*kg* _IL_ *^b^*	*g* _CO_2__/*g* _IL_ *^b^*	Ref.
[apbim][BF_4_]	22	1	269.1	~0.5	~1.86	~0.08	[22]
[aemim][BF_4_]	30	1	213.0	0.41	1.92	0.08	[27]
[Bmim][Met]	25	2	287.4	0.42	1.46	0.06	[30]
[Bmim][Pro]	25	2	253.3	0.32	1.26	0.06	[30]
[PEG_150_MeBu_2_NLi][Tf_2_N]	25	1	562.5	0.66	1.17	0.05	[31]
[PEG_150_MeTMGLi][Tf_2_N]	25	1	548.5	0.89	1.62	0.07	[31]
DAIL *^c^*	30	1	249.2	1.05	4.21 (0.42)	0.19 (0.02)	[32]
[TETA][NO_3_] *^d^*	15	1	209.3	1.49	7.12 (2.85)	0.31 (0.13)	[34]
[Li(HDA)][Tf_2_N]	40	1	391.2	0.88	2.25	0.10	[35]
[Li(DOBA)][Tf_2_N]	40	1	435.3	0.90	2.07	0.09	[35]
[Li(TEPA)][Tf_2_N]	80	0.1	476.4	0.72	1.51	0.07	[36]
[Li(TEPA)][Tf_2_N] *^e^*	80	0.1	476.4	1.95	4.09 (1.36)	0.18 (0.06)	[36]

*^a^* Molecular weight of pure IL. *^b^* Values shown in brackets are based on the total weight of IL + support or IL + solvent. *^c^* Mixed with H_2_O (mass ratio of IL: H_2_O is 10:90). *^d^* Mixed with H_2_O (mass ratio of IL: H_2_O is 40:60). *^e^* Mixed with [Li(TEG)][Tf_2_N] (mass ratio is 1:2).

**Table 2:** Typical anion-functionalized ILs for CO_2_ capture via single-site mechanisms.

IL	*T* (°C)	*P* (bar)	*M*_w_ (g mol^–1^) *^a^*	*n* _CO_2__/*n* _IL_	*n* _CO_2__/*kg* _IL_ *^b^*	*g* _CO_2__/*g* _IL_ *^b^*	Ref.
[P_4444_][Gly] *^c^*	–	1	333.5	~0.6	~1.80 (0.74)	~0.08 (0.03)	[38]
[P_4444_][Ala] *^c^*	–	1	347.5	~0.67	~1.93 (0.81)	~0.08 (0.04)	[38]
[P_4444_][β-Ala] *^c^*	–	1	347.5	~0.6	~1.73 (0.72)	~0.08 (0.03)	[38]
[P_66614_][Met]	22	1	632.1	~0.9	~1.42	~0.06	[39]
[P_66614_][Pro]	22	1	598.0	~0.9	~1.51	~0.07	[39]
[P_4444_][Butyrate]	40	1	346.5	0.4	1.15	0.05	[51]
[MTBDH][TFE]	23	1	253.3	1.13	4.46	0.20	[57]
[MTBDH][TFPA]	23	1	329.4	0.93	2.82	0.12	[57]
[MTBDH]_2_[HFPD]	23	1	518.5	2.04	3.93	0.17	[57]
[DBUH][TFE]	r.t.	1	252.3	1.01	4.00	0.18	[58]
[P_66614_][4-Me-PhO]	30	1	591.0	0.91	1.54	0.07	[59]
[P_66614_][4-H-PhO]	30	1	577.0	0.85	1.47	0.06	[59]
[P_66614_][4-Cl-PhO]	30	1	611.4	0.82	1.34	0.06	[59]
[P_66614_][4-CF_3_-PhO]	30	1	645.0	0.61	0.95	0.04	[59]
[P_66614_][4-NO_2_-PhO]	30	1	622.0	0.30	0.48	0.02	[59]
[P_66614_][2,4,6-Cl-PhO]	30	1	680.3	0.07	0.10	0.0044	[59]
[P_66614_][4-Kt-PhO]	30	1	619.0	1.04	1.68	0.07	[60]
[P_66614_][4-EF-PhO]	30	1	649.0	1.03	1.59	0.07	[60]
[P_66614_][4-CHO-PhO]	30	1	605.0	1.01	1.67	0.07	[60]
[P_66614_][PPhO]	20	1	653.1	0.93	1.42	0.06	[61]
[P_66614_][PCCPhO]	20	1	679.1	0.96	1.41	0.06	[61]
[P_4444_][2-F-PhO]	40	1	370.5	0.67	1.81	0.08	[62]
[P_4444_][3-F-PhO]	40	1	370.5	0.74	2.00	0.09	[62]
[P_4444_][4-F-PhO]	40	1	370.5	0.84	2.27	0.10	[62]
[Na(15-crown-5)][PhO]	25	1	336.4	0.75	2.23	0.10	[64]
[Na(15-crown-5)][*n*-C_3_H_7_PhO]	25	1	378.4	0.66	1.74	0.08	[64]
[Na(15-crown-5)][*n*-C_8_H_17_PhO]	25	1	448.6	0.50	1.11	0.05	[64]
[MTBDH][Im]	23	1	221.3	1.03	4.65	0.20	[57]
[(P_2_-Et)H][Im]	23	1	407.5	0.96	2.36	0.10	[57]
[P_66614_][Im]	23	1	550.9	1.00	1.82	0.08	[65]
[TMGH][Im]	30	1	183.3	1.00	5.46	0.24	[67]
[DBUH][Im]	25	1	220.3	~0.88	~3.99	~0.18	[69]
[DBNH][Im]	25	1	192.3	0.8	4.16	0.18	[70]
[DMAPAH][Im]	22	1	170.3	0.81	4.76	0.21	[72]
[DMEDAH][Im]	22	1	156.2	0.77	4.93	0.22	[73]
[P_66614_][Pyrz]	23	1	550.9	1.02	1.85	0.08	[65]
[P_66614_][Tetz]	23	1	552.9	0.08	0.14	0.01	[65]
[P_66614_][Triz]	23	1	551.9	0.95	1.72	0.08	[65]
[P_66614_][4-CHO-Im]	20	1	578.9	1.24	2.14	0.09	[60]
[P_66614_][4-Br-Im]	20	1	629.8	0.87	1.38	0.06	[66]
[P_66614_][2-CN-Pyr]	22	1	575.0	0.9	1.57	0.07	[82]
[P_66614_][2-CF_3_-Pyra]	22	1	618.9	0.9	1.45	0.06	[82]

*^a^* Molecular weight of pure IL. *^b^* Values shown in brackets are based on the total weight of IL + support or IL + solvent. *^c^* Immobilization of IL on porous silica gel (SiO_2_) support (molar ratio of IL: SiO_2_ is 1:8).

**Table 3:** Typical anion-functionalized ILs for CO_2_ capture via multiple-site mechanisms.

IL	*T* (°C)	*P* (bar)	*M*_w_ (g mol^–1^)	*n* _CO_2__/*n* _IL_	*n* _CO_2__/*kg* _IL_	*g* _CO_2__/*g* _IL_	Ref.
[P_66614_][Lys]	22	1	629.0	1.37	2.18	0.10	[101]
[N_66614_][Lys]	22	1	612.1	2.1	3.43	0.15	[102]
[C_2_OHmim][Lys]	30	1	272.3	1.68	6.17	0.27	[103]
[N_1,1,6,2O4_][Lys]	20	1	375.6	1.62	4.31	0.19	[104]
[N_66614_][His]	22	1	612.0	1.9	3.10	0.14	[102]
[N_66614_][Asn]	22	1	598.0	2.0	3.34	0.15	[102]
[N_66614_][Gln]	22	1	612.1	1.9	3.10	0.14	[102]
[MTBDH]_2_[HFPD]	23	1	518.5	2.04	3.93	0.17	[57]
[P_66614_]_10_[DCP5]	50	1	6019.5	5.52	0.92	0.04	[108]
[P_66614_][2-Op]	20	1	578.0	1.58	2.73	0.12	[110]
[P_66614_][4-Op]	20	1	578.0	1.49	2.58	0.11	[110]
[P_66614_][3-Op]	20	1	578.0	1.38	2.39	0.11	[110]
[P_4442_][2-Op]	30	1	325.5	1.40	4.30	0.19	[112]
[N_4442_][2-Op]	30	1	308.5	1.24	4.02	0.18	[112]
[Bmim][2-Op]	30	1	233.3	1.02	4.37	0.19	[112]
[P_4442OH_][2-Op]	30	1	341.5	0.94	2.75	0.12	[112]
[Ph-C_8_eim][2-Op]	30	1	379.5	1.69	4.45	0.20	[112]
[Ph-C_8_eim][2-Op]	20	1	379.5	1.83	4.82	0.21	[112]
[DBUH][2-Op]	40	1	247.3	~0.86	~3.48	~0.15	[113]
[TMGH][2-Op]	40	1	210.3	~0.82	~3.90	~0.17	[113]
[P_4442_][Suc]	20	1	329.5	1.87	5.68	0.25	[117]
[P_4442_][Suc]	20	0.1	329.5	1.65	5.01	0.22	[117]
[P_4442_][DAA]	20	1	331.5	1.25	3.77	0.17	[117]
[P_4442_][DAA]	20	0.1	331.5	1.12	3.38	0.15	[117]
[P_4442_][Cy-Suc]	20	1	383.6	2.21	5.76	0.25	[118]
[P_4442_][Ph-Suc]	20	1	377.5	1.0	2.65	0.12	[118]
[P_66614_][MA-Tetz]	30	1	581.9	1.13	1.94	0.09	[121]
[P_4442_]_2_[IDA]	40	1	593.8	1.69	2.85	0.13	[122]
[P_4442_]_2_[D-Ser]	25	1	565.8	1.06	1.87	0.08	[123]
[P_4442_]_2_[L-Ser]	25	1	565.8	1.10	1.94	0.09	[123]
[P_66614_]_2_[AA-Su]	30	1	1148.9	1.48	1.29	0.06	[124]
[P_66614_]_2_[AA-Ac]	30	1	1112.8	1.97	1.77	0.08	[124]
[P_66614_]_2_[AA-Im]	30	1	1120.9	1.55	1.38	0.06	[124]
[P_66614_]_2_[AA-Ind]	30	1	1169.9	1.45	1.24	0.05	[124]
[P_4444_]_3_[2,4-OPym-5-Ac]	r.t.	1	931.4	1.46	1.57	0.07	[125]
[P_66614_]_2_[Am-iPA]	30	1	1146.8	2.38	2.08	0.09	[124]

**Table 4:** Typical cation-anion dual-functionalized ILs for CO_2_ capture.

IL	*T* (°C)	*P* (bar)	*M*_w_ (g mol^–1^) *^a^*	*n* _CO_2__/*n* _IL_	*n* _CO_2__/*kg* _IL_ *^b^*	*g* _CO_2__/*g* _IL_ *^b^*	Ref.
[aP_4443_][Gly] *^c^*	–	1	334.5	~0.94	~2.81 (1.15)	~0.12 (0.05)	[128]
[aP_4443_][Ala] *^c^*	–	1	348.5	~0.92	~2.64 (1.11)	~0.12 (0.05)	[128]
[aemmim][Tau]	30	1	264.4	~0.9	~3.40	~0.15	[129]
[apaeP_444_][Lys] *^d^*	25	1	448.7	1.73	3.86 (1.93)	0.17 (0.08)	[130]
[apaeP_444_][Gly] *^d^*	25	1	377.6	1.29	3.42 (1.71)	0.15 (0.08)	[130]
[apaeP_444_][Ser] *^d^*	25	1	407.6	1.19	2.92 (1.46)	0.13 (0.06)	[130]
[apaeP_444_][Ala] *^d^*	25	1	391.6	1.14	2.91 (1.46)	0.13 (0.06)	[130]
[apaeP_444_][Asp] *^d^*	25	1	435.6	1.07	2.46 (1.23)	0.11 (0.05)	[130]
[apaeP_444_][His] *^d^*	25	1	457.6	1.01	2.21 (1.11)	0.10 (0.05)	[130]
[AEMP][Gly] *^e^*	–	1	218.3	1.50	6.87 (1.37)	0.30 (0.06)	[131]
[AEMP][Ala] *^e^*	–	1	232.3	1.57	6.76 (1.35)	0.30 (0.06)	[131]
[AEMP][Pro] *^e^*	–	1	258.4	1.54	5.96 (1.19)	0.26 (0.05)	[131]
[AEMP][Leu] *^e^*	–	1	274.4	1.47	5.36 (1.07)	0.24 (0.05)	[131]
[APmim][Gly] *^f^*	30	1	214.3	1.23	4.31	0.19	[133]
[APmim][Lys] *^f^*	30	1	285.4	1.80	6.71	0.27	[132]
[TETAH][Lys] *^f^*	40	1	292.4	2.59	8.86	0.39	[134]
[DETAH][Lys] *^f^*	40	1	249.4	2.13	8.55	0.38	[134]
[aP_4443_][2-Op]	30	1	354.5	1.57	4.44	0.20	[135]
[aP_4443_][2-Np]	30	1	353.5	1.88	5.32	0.23	[135]
[aP_4443_][Triz]	30	1	328.5	1.32	4.02	0.18	[135]
[Na(MDEA)_2_][Pyrz]	80	1	328.4	0.75	2.28	0.10	[137]
[K(DGA)_2_][Im]	60	1	316.4	1.37	4.33	0.19	[138]
[K(AMP)_2_][Im]	60	1	284.4	1.19	4.18	0.18	[139]

*^a^* Molecular weight of pure IL. *^b^* Values shown in brackets are based on the total weight of IL + support or IL + solvent. *^c^* Immobilization of the IL on porous SiO_2_ support (molar ratio of IL: SiO_2_ is 1:8), and absorption by SiO_2_ is subtracted. *^d^* Immobilization of the IL on porous SiO_2_ support (mass ratio of IL: SiO_2_ is 1:1). *^e^* Immobilization of the IL on porous SiO_2_ support (mass ratio of IL: SiO_2_ is 1:4). *^f^* Mixed with H_2_O (IL concentration: 0.5 mol L^−1^).

**Table 5:** The viscosities of typical functionalized ILs before and after CO_2_ capture.

IL	*T* (°C)	*P* (bar)	Viscosity of IL (cP)	Viscosity of IL + CO_2_ (cP)	Viscosity Increase (fold)	Ref.
[APbim][BF_4_]	22	1	-	-	Dramatic increase	[22]
[P_66614_][Pyr]	23	1	245.4	555.1	2.26	[65]
[P_66614_][Oxa]	23	1	555.5	1145.8	2.06	[65]
[P_66614_][PhO]	23	1	390.3	645.4	1.65	[65]
[P_66614_][Im]	23	1	810.4	648.7	0.84	[65]
[P_66614_][2-CN-Pyr]	25	1	360	370	1.03	[82]
[P_66614_][3-CF_3_-Pyr]	25	1	270	500	1.85	[82]
[P_66614_][Pro]	20	1	1000	1700	1.7	[101]
[P_66614_][Met]	25	1	350	33,000	94	[101]
[P_66614_][Lys]	20	1	1000	280,000	280	[101]
[P_66614_][Tau]	25	1	670	44,000	66	[101]
[P_66614_][2-Op]	20	1	573	2273	4	[110]
[P_4442_][Suc]	20	0.1	998	629	0.63	[117]
[P_4442_][DAA]	20	0.1	605	147	0.24	[117]
[P_4442_]_2_[IDA]	40	1	66.2	961.6	14.5	[122]
[aP_4443_][Gly]	25	1	713.9	-	Dramatic increase	[128]
[Na(MDEA)_2_][Pyrz]	50	1	1310	713.9	0.54	[137]

## Data Availability

Not applicable.
